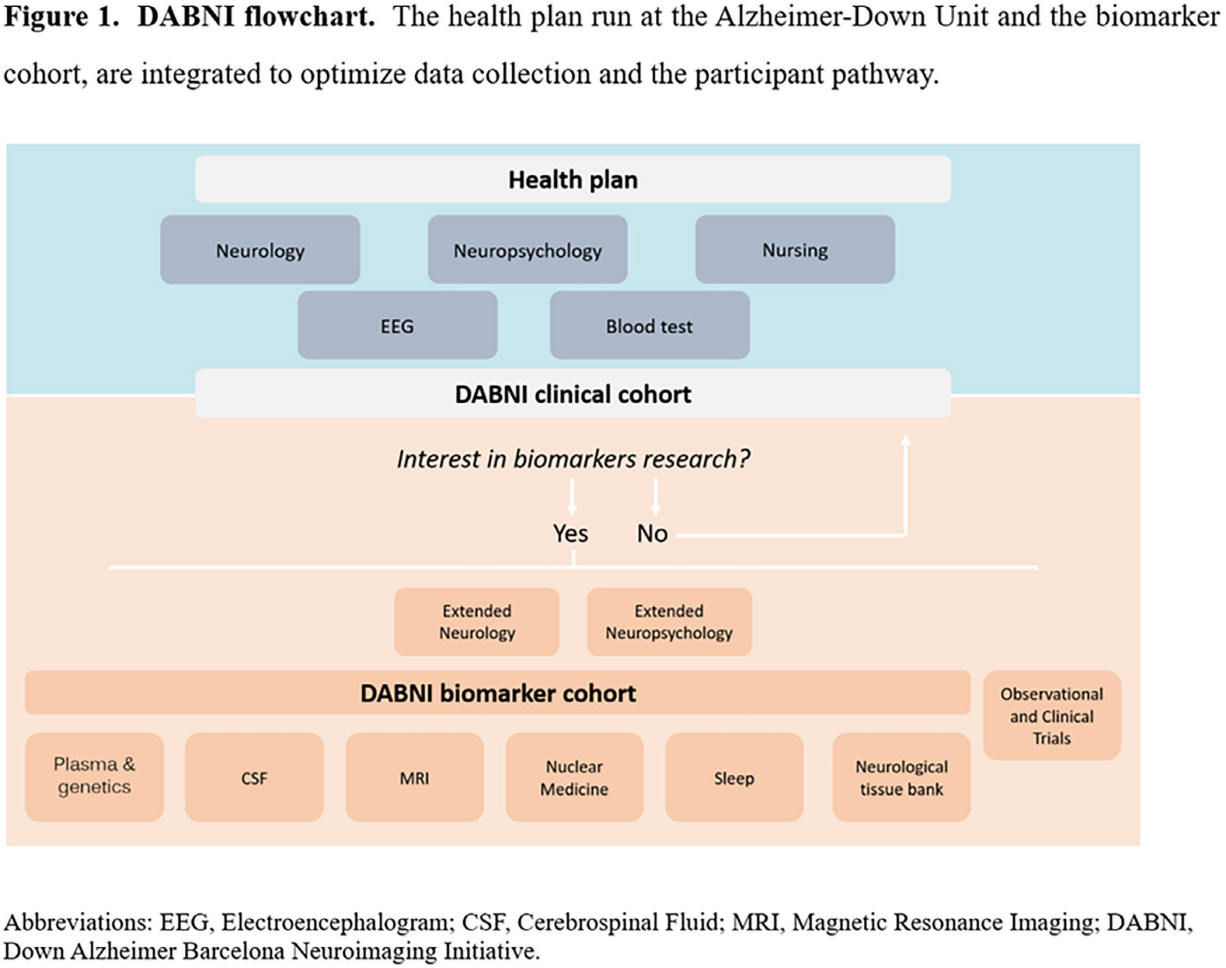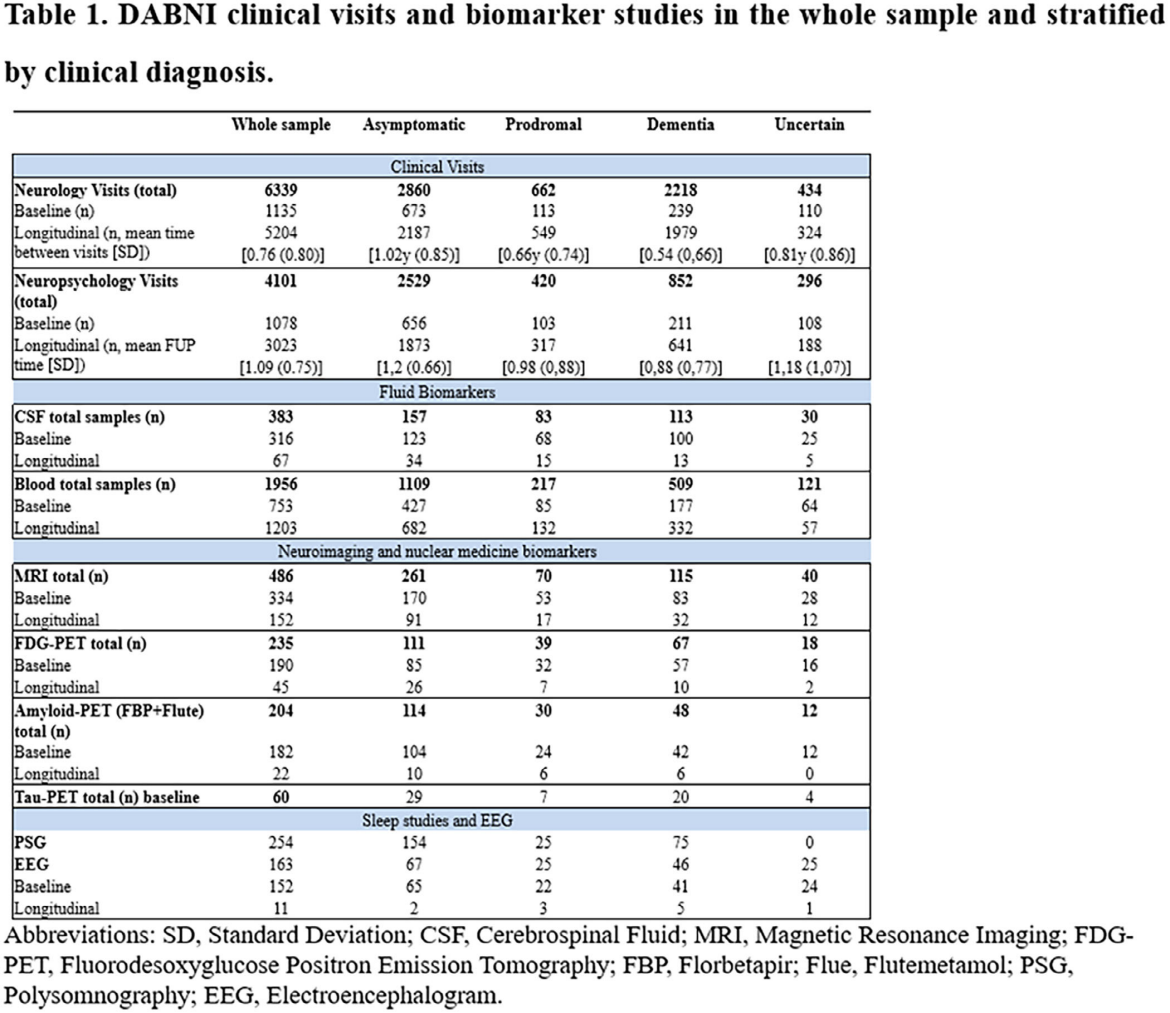# The Down Alzheimer Barcelona Neuroimaging Initiative (DABNI): Ten Years of Progress in Understanding Alzheimer's in Down Syndrome

**DOI:** 10.1002/alz70856_106426

**Published:** 2026-01-10

**Authors:** Laura Videla, Bessy Benejam, Isabel Barroeta, Susana Fernandez, Javier Arranz, Íñigo Rodríguez‐Baz, José Enrique Arriola‐Infante, Lucía Maure‐Blesa, Laura Del Hoyo, Lídia Vaqué‐Alcázar, Mateus Rozalem Aranha, Alejandra O. Morcillo‐Nieto, Sara E Zsadanyi, Valle Camacho, Sandra Giménez, Olivia Belbin, Daniel Alcolea, Alexandre Bejanin, Sebastián Videla, Rafael Blesa, Alberto Lleó, Maria Carmona‐Iragui, Juan Fortea

**Affiliations:** ^1^ CIBERNED, Network Center for Biomedical Research in Neurodegenerative Diseases, National Institute of Health Carlos III, Madrid, Spain; ^2^ Sant Pau Memory Unit, Hospital de la Santa Creu i Sant Pau, Institut de Recerca Sant Pau ‐ Universitat Autònoma de Barcelona, Barcelona, Spain; ^3^ Barcelona Down Medical Center, Fundació Catalana Síndrome de Down, Barcelona, Spain; ^4^ Center for Biomedical Investigation Network for Neurodegenerative Diseases (CIBERNED), Madrid, Spain; ^5^ Centro de Investigación Biomédica en Red en Enfermedades Neurodegenerativas, CIBERNED, Barcelona, Spain; ^6^ Sant Pau Memory Unit, Hospital de la Santa Creu i Sant Pau, Biomedical Research Institute Sant Pau, Universitat Autònoma de Barcelona, Barcelona, Spain; ^7^ Department of Medicine, Faculty of Medicine and Health Sciences, Institute of Neurosciences, University of Barcelona, Barcelona, Spain. Institut d’Investigacions Biomèdiques August Pi i Sunyer (IDIBAPS), Barcelona, Spain; ^8^ Neuroradiology Section, Department of Radiology, Hospital de la Santa Creu i Sant Pau, Biomedical Research Institute Sant Pau, Universitat Autònoma de Barcelona, Spain, Barcelona, Spain; ^9^ Center of Biomedical Investigation Network for Neurodegenerative Diseases (CIBERNED), Madrid, Spain; ^10^ Nuclear Medicine Department, Hospital de la Santa Creu i Sant Pau, Barcelona, Barcelona, Spain; ^11^ Multidisciplinary Sleep unit. Hospital de la Santa Creu i Sant Pau, Institut d'Investigació Biomèdica Sant Pau (IIB SANT PAU), Barcelona, Spain; ^12^ Clinical Research Support Unit. Bellvitge Biomedical Research Institute (IDIBELL) Department of Clinical Pharmacology, University of Barcelona, Barcelona, Spain; ^13^ Sant Pau Memory Unit, Department of Neurology, Hospital de la Santa Creu i Sant Pau, Institut d'Investigació Biomèdica Sant Pau (IIB SANT PAU), Facultad de Medicina ‐ Universitat Autònoma de Barcelona, Barcelona, Spain; ^14^ Centre Médic Down de la Fundació Catalana Síndrome de Down, Barcelona, Spain; ^15^ Centre of Biomedical Investigation Network for Neurodegenerative Diseases (CIBERNED), Madrid, Spain

## Abstract

**Background:**

Down syndrome (DS) is a form of genetically determined Alzheimer's disease (AD). Understanding the natural history and biomarker specificities in this population can offer insights into AD pathogenesis and potential treatment targets.

**Methods:**

The Down Alzheimer Barcelona Neuroimaging Initiative (DABNI) is a prospective longitudinal cohort of adults with DS that builds on a population‐based health plan screening for neurological conditions (Figure 1). Conducted at the Alzheimer‐Down Unit, the study aims to elucidate the natural history of AD in DS through multimodal biomarker investigations and support clinical trials. Participants undergo neurological and neuropsychological evaluations, blood and cerebrospinal fluid collection, structural 3T brain MRI, PET imaging ([18F]FDG‐PET, amyloid‐PET or tau‐PET), as well as electroencephalography and video‐polysomnography examinations.

**Results:**

The DABNI cohort included 1,135 participants with a mean age of 42.82 years (SD = 11.56), 46.3% of whom are female. At baseline, 673 participants were asymptomatic or cognitively stable, 113 had prodromal AD, 239 had AD dementia, and 110 were uncertain due to non‐AD conditions. The cohort's intellectual disability level distribution was 20% mild, 50% moderate, 20% severe, and 7% profound. Over 10,000 visits have demonstrated that AD progression is rare before age 40, but high thereafter (57.5% of individuals over 50 progress within 5 years). Neuropsychological assessments and biomarkers have shown strong diagnostic performance for symptomatic AD, and a prolonged and predictable preclinical phase similar to autosomal dominant AD. The initiative is actively participating in three clinical trials and has yielded a robust body of work, with nearly 100 publications. Table 1 shows the number of clinical visits and biomarker studies in the whole sample and stratified by clinical diagnosis.

**Conclusion:**

In 10 years, the DABNI study has become one of the largest multimodal initiatives on AD in adults with DS and has provided critical insights into disease progression and biomarkers, advancing interventions and clinical research